# *Bacillus subtilis* and *Pseudomonas fluorescens* Trigger Common and Distinct Systemic Immune Responses in *Arabidopsis thaliana* Depending on the Pathogen Lifestyle

**DOI:** 10.3390/vaccines8030503

**Published:** 2020-09-04

**Authors:** Ngoc Huu Nguyen, Patricia Trotel-Aziz, Sandra Villaume, Fanja Rabenoelina, Adrian Schwarzenberg, Eric Nguema-Ona, Christophe Clément, Fabienne Baillieul, Aziz Aziz

**Affiliations:** 1 Induced Resistance and Plant Bioprotection EA 4707, SFR Condorcet FR-CNRS 3417, UFR Sciences, Campus Moulin de la Housse, University of Reims, CEDEX 02, 51687 Reims, France; huunguyentnu@gmail.com (N.H.N.); patricia.trotel-aziz@univ-reims.fr (P.T.-A.); sandra.villaume@univ-reims.fr (S.V.); clarisse.rabenoelina@univ-reims.fr (F.R.); christophe.clement@univ-reims.fr (C.C.); fabienne.baillieul@univ-reims.fr (F.B.); 2 Centre Mondial de l’Innovation, Groupe Roullier, 35401 Saint-Malo, France; a.schwarzenberg@yahoo.fr (A.S.); Eric.NguemaOna@roullier.com (E.N.-O.)

**Keywords:** *Arabidopsis*, *Bacillus subtilis*, induced immunity, pathogens, priming, *Pseudomonasfluorescens*, induced resistance

## Abstract

Plants harbor various beneficial bacteria that modulate their innate immunity, resulting in induced systemic resistance (ISR) against various pathogens. However, the immune mechanisms underlying ISR triggered by Bacillus spp. and Pseudomonas spp. against pathogens with different lifestyles are not yet clearly elucidated. Here, we show that root drenching of Arabidopsis plants with *Pseudomonas fluorescens* PTA-CT2 and *Bacillus subtilis* PTA-271 can induce ISR against the necrotrophic fungus *B. cinerea* and the hemibiotrophic bacterium *Pseudomonas syringae Pst* DC3000. In the absence of pathogen infection, both beneficial bacteria do not induce any consistent change in systemic immune responses. However, ISR relies on priming faster and robust expression of marker genes for the salicylic acid (SA), jasmonic acid (JA), and ethylene (ET) signaling pathways upon pathogen challenge. These responses are also associated with increased levels of SA, JA, and abscisic acid (ABA) in the leaves of bacterized plants after infection. The functional study also points at priming of the JA/ET and NPR1-dependent defenses as prioritized immune pathways in ISR induced by both beneficial bacteria against *B. cinerea*. However, *B. subtilis*-triggered ISR against *Pst* DC3000 is dependent on SA, JA/ET, and NPR1 pathways, whereas *P. fluorescens*-induced ISR requires JA/ET and NPR1 signaling pathways. The use of ABA-insensitive mutants also pointed out the crucial role of ABA signaling, but not ABA concentration, along with JA/ET signaling in primed systemic immunity by beneficial bacteria against *Pst* DC3000, but not against *B. cinerea*. These results clearly indicate that ISR is linked to priming plants for enhanced common and distinct immune pathways depending on the beneficial strain and the pathogen lifestyle.

## 1. Introduction

In hostile habitats, plants are subjects to various pathogens, including necrotrophs, biotrophs, and hemibiotrophs [1]. To restrict the pathogen infection, plants rapidly activate different layers of defenses depending on its own ability to recognize conserved microbial molecules that are characteristic of microorganisms [2,3]. The recognition of these microbial molecules known as “microbe-associated molecular patterns” (MAMPs) is mediated by a set of receptors referred to as pattern-recognition receptors (PRRs). Once pathogen or non-pathogen microbes are perceived by the plant, immune responses are often triggered in distal plant parts to protect undamaged tissues against subsequent pathogen infection. This long-lasting induced immunity is referred to as systemic acquired resistance (SAR) or induced systemic resistance (ISR) [4,5]. Accumulating evidence has shown that treatment of plant roots with beneficial bacteria enhance plant health through ISR [5], that mostly linked to a stronger and faster systemic immune response after pathogen infection, a phenomenon known as priming that allows plants to alert their immune system, reducing then their energy consumption [6,7]. The beneficial bacteria *Bacillus* spp. and *Pseudomonas* spp. have been reported to elicit ISR against pathogens with different lifestyles in various plant species through priming phenomenon [8,9,10,11,12,13,14]. The primed plants express upregulation of defense related genes [15,16,17], generation of reactive oxygen species [18], reinforcement of cell walls via callose deposition [8,13], and accumulation of anti-microbial phytoalexins [11,19] after perception of pathogen-derived signal.

Salicylic acid (SA), jasmonate (JA), and ethylene (ET) have been documented as the major phytohormones involved in regulation of host immune response to pathogens with different lifestyles [20,21]. JA and ET signaling pathways are more important for resistance to necrotrophic fungi including *Botrytis cinerea*, while SA signaling is generally effective in response to biotrophs or hemibiotrophs, such as *Pseudomonas syringae* pv. *tomato* DC3000. Collectively, the beneficial bacteria-mediated ISR seems to prioritize JA and ET signaling pathways that regulate *PDF1.2* gene expression [5,13,16,17,22,23], whereas pathogen-induced SAR involves SA accumulation and *PR1* expression [4,24]. Some beneficial microbes may also mediate ISR in SA-dependent manner and priming of *PR1* gene expression upon pathogen challenge. For example, the *Bacillus cereus* AR156-mediated ISR against *Pst* DC3000 is simultaneously dependent on the activation of SA-responsive genes *PR1*, *PR2*, *PR5*, and JA/ET-responsive gene *PDF1.2* [16]. *Pseudomonas fluorescens* SS101-mediated ISR against *Pst* DC3000 was impaired in SA deficient *NahG* plants, but remained comparable in JA- and ET-insensitive *jar1* and *ein2* mutants, respectively, compared to the wild type Col-0 [25]. The beneficial bacterium *Bacillus subtilis* FB17 also triggers ISR in Arabidopsis in a SA and ET dependent manner [26]. This revealed complex regulatory networks in the hormone signaling sectors triggered by different beneficial bacteria, that might affect the efficiency of ISR against pathogens.

More recently, abscisic acid (ABA) has also been identified as a regulator of plant immunity against pathogens [27,28,29,30]. ABA can also interact with SA, JA, and ET-regulated defenses, thereby modulating pathogen resistance [31,32,33]. ABA is involved in regulation of stomatal aperture, callose deposition, or expression of defense-related genes to restrict pathogen penetration [29,34,35]. It has also been reported that ABA-deficient mutants, *aba3-1*, *aba2-1*, failed to reduce stomatal aperture after pretreatment with *B. subtilis* FB17 inducing ISR [28] The impairment of stomatal closure was also observed in *B. amyloliquefaciens* FZB42-treated ABA-insensitive mutant *abi1* [30]. The positive role of ABA in JA synthesis and induction of JA-responsive genes was also reported in Arabidopsis during interaction with the vascular oomycete *Pythium irregular* [36]. Moreover, priming defense response by β-aminobutyric acid (BABA) requires an ABA-dependent callose deposition upon pathogen challenge [27,37,38]. ABA has also been described as a negative regulator at the post-invasive penetration of pathogens through phytohormonal crosstalk [39,40]. Exogenous application of ABA leads to high susceptibility of Arabidopsis to *Pst* DC3000, which seems due to a negative effect on some defenses, such as inhibition of lignin biosynthesis, suppression of SA- and ET/JA-responsive genes [39,40].

In our previous research, we showed that both *Bacillus subtilis* PTA-271 and *Pseudomonas fluorescens* PTA-CT2 [41], trigger ISR in grapevine by priming plant immunity [11,18,19,42,43]. Nevertheless, whether priming is regulated by common or distinct immune pathways in *B. subtilis*- and *P. fluorescens*-mediated ISR against pathogens with different lifestyles remains to be elucidated. In this study, we first investigated the capacity of *B. subtilis* PTA-271 and *P. fluorescens* PTA-CT2 to trigger ISR against the necrotrophic fungus *B. cinerea* and the hemibiotrophic bacterium *Pst* DC3000 in *Arabidopsis thaliana*. We then examined the differences and similarities of *B. subtilis* and *P. fluorescens*-induced priming immune response and analyzed signaling pathways involved in ISR against pathogen infection. We especially focused on the expression of defense-related genes, phytohormone amounts, and the roles of SA, JA/ET, NPR1, and ABA signaling pathways in *P. fluorescens*- and *B. subtilis*-mediated ISR against *B. cinerea* and *Pst* DC3000.

## 2. Materials and Methods

### 2.1. Plant and Microbial Growth Conditions

*Arabidopsis thaliana* ecotype Col-0 and its transgenic and mutant lines, including NahG (salicylic acid-deficient; [44], sid2 (SA synthesis-difficient2; [45], npr1 (SA-insensitive, nonexpressor of PR1; [46]), ein2.1 (ethylene-insensitive2.1), jar1.2 (jasmonic acid-insensitive1.2), and ABA insensitive mutants abi1.1 and abi2.1 [47] were obtained from the Salk Institute [48]. Seeds were sterilized in 50% sodium hypochlorite solution containing 0.2% Tween 20 for 10 min, rinsed five times with sterile distilled water, and kept at 4 °C for four days to promote uniform germination. The sterilized seeds were sown on soil under controlled conditions with 8 h light/16 h dark regime at a constant temperature of 22 °C. Two-week-old soil-grown seedlings were then transferred to individual 60-mL pots for 1 week before bacterial treatment.

*B. subtilis* PTA-271 (GenBank Nucleotide Accession No. AM293677) and *P. fluorescens* PTA-CT2 (GenBank Nucleotide Accession No. AM29367), isolated from grapevine plants [41] were cultivated overnight in sterile Luria Bertani (LB) liquid medium at 28 °C, under continuous shaking at 110 rpm. The bacterial pellets were then collected at the exponential growth phase by centrifugation at 4 °C, 5000 rpm for 20 min. The bacterial pellets were washed once and resuspended in sterilized 10 mM MgSO_4_, then adjusted to 1 × 10^9^ CFU/mL for plant treatment.

*Pseudomonas syringae* pv. *tomato* DC3000 (*Pst* DC3000) was grown overnight in sterile King Broth (KB) medium containing 50 mg/L rifampicin at 28 °C under continuous shaking at 180 rpm, then the pellets were collected at the exponential growth phase by centrifugation at 4 °C, 5000 rpm for 20 min. The pellets were then washed, resuspended in sterilized 10 mM MgSO_4_ and adjusted to 1 × 10^8^ CFU/mL. *Botrytis cinerea* was grown on potato dextrose agar (PDA) at 28 °C for 2 weeks. Then fungal conidia were collected and resuspended in sterilized water, and filtrated to remove the mycelium and adjusted to 1 × 10^6^ conidia [49].

### 2.2. Bacterial Treatment, Pathogen Infection, and Disease Assessment

Three-week-old plants were soil-drenched with *B. subtilis* or *P. fluorescens* to reach a final concentration of 1 × 10^8^ CFU/g soil, or with MgSO_4_ as a mock treatment (control). Two weeks later, true rosette leaves of 5-week-old plants were infected with 10 µL droplets or by spraying *B. cinerea* at 1 × 10^6^ conidia/mL or spraying *Pst* DC3000 at 1 × 10^8^ CFU/mL. Disease symptoms were evaluated by determining the mean lesion diameter and percentage of diseased leaves in 20–30 plants per treatment at 4 days-post infection (dpi) with *B. cinerea* and *Pst* DC3000, respectively. Meanwhile, the pathogen growth was determined by analyzing the transcript level of *B. cinerea Actin* gene, and *Pst* DC3000 development was quantified on KB agar medium containing 50 mg/L rifampicin and 100 mg/L kanamycin after 48 h at 28 °C, and expressed as logarithmic scale of CFU per gram of fresh weight.

### 2.3. RNA Extraction and Analysis of Gene Expression by RT-qPCR

For each sample, 100 mg of leaves was ground in liquid nitrogen. Total RNA was isolated using Extract’ All (Eurobio, Les Ulis, France) and cDNA was then synthesized from 1 µg of total RNA followed reverse transcription by using the Verso cDNA Synthesis Kit (Thermo Fisher Scientific, USA) according to the manufacturer’s instructions. The transcript levels of target genes were determined by qPCR using the CFX 96TM Real Time System (Bio-Rad, Marnes-la-Coquette, France) and the SYBR Green Master Mix PCR kit as recommended by the manufacturer (Applied Biosystems, Foster City, CA, USA). PCR conditions were 95 °C for 15 s (denaturation) and 60 °C for 1 min (annealing/extension) for 40 cycles on CFX 96TM Real Time System (Bio-Rad, Marnes-la-Coquette, France). Traditional reference genes were evaluated with Bio-Rad CFX MANAGER software v.3.0 (*Actin2*, *UBQ5*, *UBQ10*, *EF1α*, and *Tubulin2*) to select a reference gene with a stable expression in all tested conditions. The expression stability GeNorm M value of *UBQ5* was below the critical value of 0.5 in Arabidopsis samples. Transcript levels of target genes were calculated using the standard curve method and normalized against *UBQ5* gene as an internal control. For each experiment, PCR reactions were performed in duplicate and reference samples are leaves of untreated plants as the control sample. The specific primers used in this study were listed in Table S1 in Supplementary Material.

### 2.4. Phytohormone Analysis by LC-MS

Phytohormones were extracted from 20 mg of ground leaf powder in 1 mL of cold solution of methanol/water/formic acid (70/29/1: *v/v/v*). The homogenates were stirred at room temperature for 30 min, then centrifuged at 15,000× *g* for 10 min at 4 °C. The supernatant was evaporated under nitrogen and the residue was dissolved with 1 mL of a 2% formic acid solution. The extracts were purified using a solid phase extraction (SPE) Evolute express ABN 1 mL–30 mg (Biotage, Hengoed, UK). The eluate was evaporated to dry and reconstructed in 200 µL of H_2_O containing 0.1% of formic acid.

Phytohormones, salicylic acid (SA), jasmonic acid (JA), and abscisic acid (ABA) were purchased from OlchemIn (Olomouc, Czech Republic, and the precursor of ethylene, 1-aminocyclopropane carboxylic acid (ACC) was purchased from Sigma-Aldrich (Saint-Quentin-Fallavier, France). Phytohormones and ACC were analyzed by an UHPLC-MS/MS system as described in Lakkis et al. [11]. The analysis was achieved by a Nexera X2 UHPLC system (Shimadzu, Kyoto, Japan) coupled to a QTrap 6500+ MS (Sciex, ON, Canada) equipped with an IonDrive™ turbo V electrospray (ESI) source. Then, 2 µL of purified extract were injected into a Kinetex Evo C18 core-shell column (100 × 2.1 mm, 2.6 µm, Phenomenex, Torrance, CA, USA) heated at 40 °C. Compounds were eluted using Milli-Q water (solvent A) and acetonitrile LCMS grade (Fisher Optima, Neots, UK) (solvent B), both containing 0.1% formic acid (LCMS grade) with a flow rate of 0.7 mL min^−1^. The gradient elution started with 1% B, 0.0–5.0 min 60% B, 5.0–5.5 min 100% B, 5.5–7.0 min 100 % B, 7.0–7.5 min 1% B, and 7.5–9.5 min 1% B. The ionization voltage was set to 5 kV for positive mode and −4.5 kV for negative mode producing mainly [M + H]+ and [M − H]−, respectively. The analysis was performed in scheduled MRM mode in positive and negative mode simultaneously with a polarity switching of 5 ms. Quantification was processed using MultiQuant software v3.0.2 (Sciex, ON, Canada).

### 2.5. Exogenous Application of ABA

Four-week-old Arabidopsis Col-0 was treated with 2% ethanol (control) or 100 µM ABA solution in 2% ethanol at the root level for 48 h. Then the leaves of control and ABA-treated plants were sprayed with *Pst* DC3000 at 1 × 10^8^ CFU/mL or drop-inoculated with 5 µL of *B. cinerea* at 1 × 10^6^ conidia/mL. The induced resistance was evaluated through disease incidence and disease severity measurement at four days-post infection as described below.

## 3. Results

### 3.1. B. subtilis and P. fluorescens Trigger ISR against Both B. cinerea and Pst DC3000

**Figure 1:**
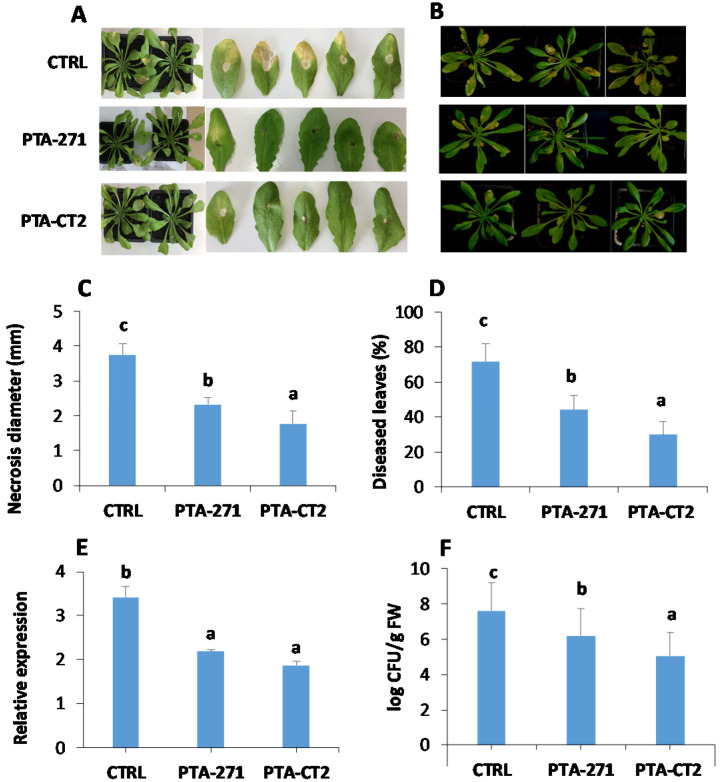
*Bacillus subtilis* PTA-271 and *Pseudomonas fluorescens* PTA-CT2 induce systemic resistance against *B. cinerea* and *Pst* DC3000 in *Arabidopsis.* Plants were treated with each bacterium at 10^8^ CFU g^−1^ soil or MgSO_4_ (control) at the root level for 2 weeks, then leaves were infected with *B. cinerea* or *Pst* DC3000. (**A**,**B**) Representative photographs depicting symptoms caused by *B. cinerea* and *Pst* DC3000 at 4 dpi. (**C**) *B. cinerea* disease incidence as the average of necrosis diameter spreading. (**D**) *Pst* DC3000 disease incidence as the percentage of diseased leaves. (**E**,**F**) The growth of *B. cinerea* and *Pst* DC3000 evaluated through the expression of *B. cinerea Actin* gene and logarithmic scale of *Pst* DC3000 CFU/g fresh weight, respectively. Data are means ± SE from at least three independent experiments with 16 plants/treatment (about 150 leaves). Different letters indicate statistically significant differences (Duncan test, *p* < 0.05).

To explore whether *B. subtilis* PTA-271 and *P. fluorescens* PTA-CT2 enable to induce systemic resistance in Arabidopsis against the necrotroph *B. cinerea* and the hemibiotroph *Pst* DC3000, 5-week-old control and bacteria-treated Col-0 plants were drop inoculated with *B. cinerea* or sprayed with *Pst* DC3000. Our results showed that *B. subtilis*- and *P. fluorescens*-treated plants displayed a significant reduction of disease symptoms provoked by *B. cinerea* (Figure 1A) and *Pst* DC3000 (Figure 1B) in the leaves. *B. subtilis-* and *P. fluorescens*-treated plants exhibited 40% and over 50% protection against *B. cinerea* and 40% and 60% protection against *Pst* DC3000, respectively, compared to control (Figure 1C,D). The expression of *B. cinerea Actin* gene was significantly reduced in the leaves of the bacteria-treated plants (Figure 1E). Similarly, the CFU quantity of *Pst* DC3000 was substantially lowered in the leaves of *B. subtilis*- and *P. fluorescens*-inoculated plants compared to control (Figure 1F). This indicates that the root treatment with *P. fluorescens* and *B. subtilis* triggers a systemic resistance in Arabidopsis against *B. cinerea* and *Pst* DC3000. The higher efficiency was observed with *P. fluorescens* compared to *B. subtilis* in inducing ISR against both pathogens.

### 3.2. B. subtilis and P. fluorescens Prime Plants for Enhanced Expression of PR1, PR4, and PDF1.2 to Different Extents after Pathogen Infection

**Figure 2:**
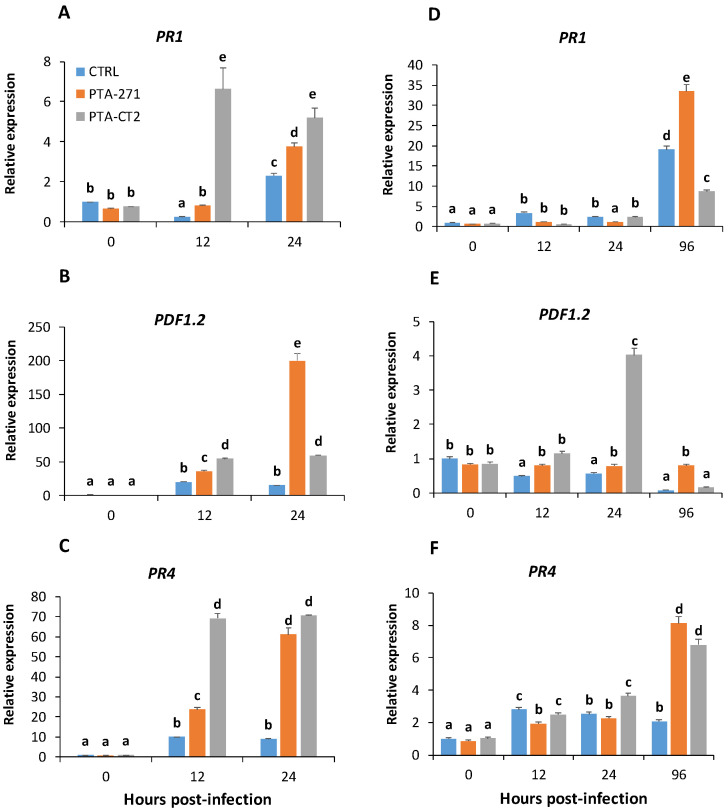
*B. subtilis* PTA-271 and *P. fluorescens* PTA-CT2 induce differential expression of defense-related genes in leaves of Col-0 plants after *B. cinerea* and *Pst* DC3000 infection. Five-week-old Col-0 were sprayed with *B. cinerea* and *Pst* DC3000, then the infected leaves were selected at 0, 12, and 24 hpi, *Pst* DC3000-infected leaves were additionally collected at 96 hpi. The *UBQ5* gene was used as internal control, and leaves without infection of control plants correspond to reference samples (assigned as 1 fold). Expression of *PR1*, *PR4*, and *PDF1.2* after *B. cinerea* (**A**–**C**) and *Pst* DC3000 (**D**–**F**) infection. The data are the mean ± SE from three independent experiments with total 12 plantlets/treatment. Different letters indicate statistically significant differences (Duncan test *p <* 0.05).

To gain more insight into the molecular mechanisms involved in *B. subtilis* and *P. fluorescens*-ISR against necrotrophic and hemibiotrophic pathogens, the transcript levels of *PR1* (SA-responsive, [50]; *PDF1.2* (ET/JA-responsive, [51] and *PR4* (ET-inducible, [52] were examined in *Arabidopsis* leaves after infection with *B. cinerea* and *Pst* DC3000. The expression of these genes was monitored in control and bacteria-treated Col-0 plants at 0, 12, and 24 h-post-infection (hpi) with *B. cinerea*, and even up to 96 hpi with *Pst* DC3000. Data (Figure 2) showed that in the absence of pathogen (0 hpi), the bacteria alone did not induce any consistent change in gene expression. However, both *P. fluorescens* and *B. subtilis* primed plants for enhanced expression of all selected genes at 12 hpi of *B. cinerea*, except *PR1* in *B. subtilis*-treated plants (Figure 2A–C). *P. fluorescens* strongly upregulated *PR1*, *PDF1.2*, and *PR4* at 12 hpi in comparison with *B. subtilis*. However, this induction remained stable or slightly reduced at 24 hpi. With *B. subtilis* these genes were upregulated later and reached the peak at 24 hpi, especially for *PDF1.2* that was approximately 4- and 10-times higher compared to *P. fluorescens*-treated and control plants, respectively (Figure 2B). The scale of *PR4* and *PDF1.2* expression was significantly higher than that of *PR1* gene after *B. cinerea* infection. The maximum transcript value of *PR1* was approximately 20- and 10-times lower than *PDF1.2* and *PR4* expression in *B. subtilis*- and *P. fluorescens*-treated plants, respectively (Figure 2A–C). These results indicate that ET and/or JA-inducible defense responses are likely to be more predominant than SA-dependent responses in resistance against *B. cinerea*. Data also revealed differential activation of gene expression by beneficial bacteria after *Pst* DC3000 infection. After bacterial treatment *PR1* induction was the same as the control at 12 hpi, and even decreased in response to *B. subtilis* at 24 hpi. However, the expression of *PR1* as for *PR4* was preferably and highly upregulated by *B. subtilis* at 96 hpi, indicating that this bacterium can initially weaken SA immune response, but later activate both SA and ET defense pathways. Interestingly, *P. fluorescens* activated the expression of *PDF1.2* and *PR4* to a high extent at 24 and 96 hpi, respectively (Figure 2D–F). Data suggest a prominent role of *PR4* in ISR against both necrotrophic and hemibiotrophic pathogens. *P. fluorescens*-treated plants activated sooner immune response to *Pst* DC3000 compared to *B. subtilis*. The expression of *PR4* was also slightly induced, while that of *PDF1.2* was about 4-times higher at 24 hpi in *P. fluorescens*-treated plants than in control (Figure 2E,F). *PDF1.2* was significantly decreased, but a significant upregulation of *PR4* was observed in *P. fluorescens*-treated plants at 96 hpi. Interestingly, the scale of *PR1* expression was stronger than *PR4* in *B. subtilis*-treated plants, indicating that SA-responsive defenses can play a crucial role in *B. subtilis*-ISR against *Pst* DC3000. Data also suggest that different mechanisms could be involved in ISR triggered by *B. subtilis* and *P. fluorescens* against the hemibiotrophic bacterium *Pst* DC3000.

### 3.3. B. subtilis and P. fluorescens Induce Differential Change in Phytohormone Amounts after Pathogen Challenge

**Figure 3:**
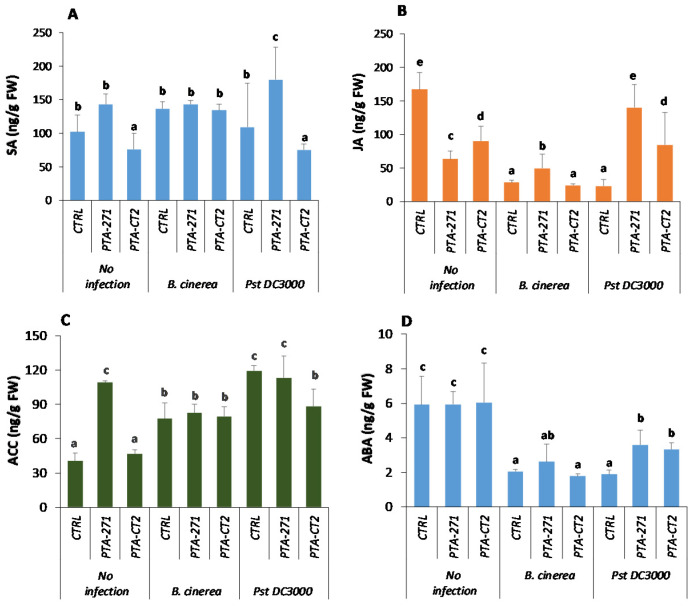
*B. subtilis* PTA-271 and *P. fluorescens* PTA-CT2 induce differential accumulation of phytohormones in Col-0 plants upon *B. cinerea* and *Pst* DC3000 challenge. Salicylic acid (**A**), jasmonic acid (**B**), 1-aminocyclopropane-carboxyliate (**C**), and abscisic acid (**D**) were quantified in control and infected leaves at 48 h-post infection of treated and non-treated plants. Data are mean ± SE from three independent experiments with a total of nine plants/treatment, each treatment with three technical repetitions. Different letters indicate statistically significant differences (Duncan test *p* < 0.05).

In order to investigate whether the expression of defense genes is associated with phytohormone accumulation at the systemic level, the amount of SA, JA, ACC (ET precursor), and ABA was quantified by LC-MS-MS in the leaves of *P. fluorescens*- and *B. subtilis*-treated plants before and after *B. cinerea* or *Pst* DC3000 infection. Data showed that in the absence of pathogen, the amounts of SA (Figure 3A) remained unchanged by *B. subtilis*, but decreased by *P. fluorescens* treatment. However, the JA content decreased in response to both bacteria (Figure 3B), while that of ABA (Figure 3D) remained stable. The ACC content (Figure 3C) increased by *B. subtilis*, but not by *P. fluorescens*. After infection with *B. cinerea*, the amount of phytohormones did not change in *P. fluorescens*-treated plants (Figure 3), whereas JA and ABA levels slightly increased in the leaves of *B. subtilis*-treated plants (Figure 3B,D). This indicates that upregulation of *PR1* induced by both bacteria is not related to enhancement of SA production upon *B. cinerea* challenge. However, after *Pst* DC3000 infection, *B. subtilis* primed plants for increased level of SA (Figure 3A), whereas JA production was induced by both bacteria but more strongly by *B. subtilis*, with approximately 2-fold higher than with *P. fluorescens* (Figure 3B). In this condition, no increase of ACC level was observed among control and bacteria-treated plants, albeit *P. fluorescens* slightly decreased ACC accumulation after *Pst* DC3000 infection (Figure 3C). Data also showed that after pathogen infection, ABA content declined from 1.5- to 2-times compared to non-infected mock. *P. fluorescens* had no significant effect on the amount of ABA compared to control plants after *B. cinerea* infection, while the ABA content increased by approximately 2-fold after *Pst* DC3000 infection (Figure 3D). This indicates that *P. fluorescens*-induced ABA synthesis depends on the pathogen lifestyle, while *B. subtilis*-treated plants accumulated ABA after infection with both pathogens.

### 3.4. B. subtilis and P. fluorescens Mediated ISR through Different Signaling Pathways

#### 3.4.1. Role of SA, JA and ET Signaling in ISR against *B. cinerea* and *Pst* DC3000

**Figure 4:**
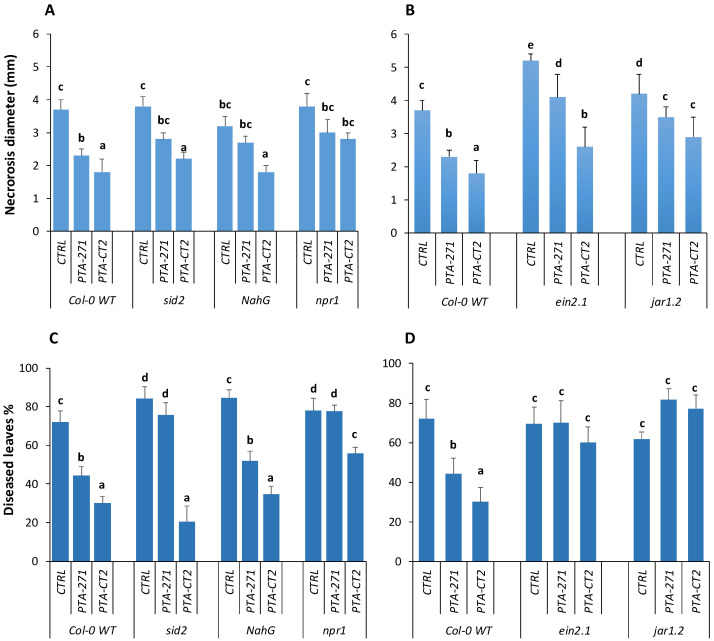
*B. subtilis* PTA-271 and *P. fluorescens* PTA-CT2-induced systemic resistance in Col-0 and its mutants *sid2*, *NahG*, *npr1*, *ein2.1*, and *jar1.2* plants against *B. cinerea* and *Pst* DC3000. Three-week-old Col-0, transgenic *NahG* and mutant plants *sid2*, *nrp1*, *ein2.1*, and *jar1.2* were pretreated with *B. subtilis* PTA-271 or *P. fluorescens* PTA-CT2 for 2 weeks. Then the leaves were infected with *B. cinerea* and *Pst* DC3000. The induced systemic resistance (ISR) was determined at 4 dpi by measuring necrosis size caused by *B. cinerea* (**A**,**B**) and the percent of diseased leaves by *Pst* DC3000 (**C**,**D**). Data are means ± SE from four independent experiments with a total of 16 plants/treatment (about 150 leaves). Different letters indicate statistically significant differences (Duncan test *p* < 0.05).

Here, we compared the effectiveness of *P. fluorescens* and *B. subtilis* in inducing ISR in the wild type Col-0 and transgenic or mutant lines *NahG*, *sid2*, *npr1*, *jar1.2*, and *ein2.1*. The results showed that *sid2* and *NahG* plants failed to fully express the ISR triggered by *B. subtilis* after *B. cinerea* infection compared to Col-0 (Figure 4A). The necrotic size caused by *B. cinerea* in the leaves of *NahG* and *sid2* was significantly larger than that in Col-0 plants, indicating that SA influences the efficiency of *B. subtilis*-ISR against this fungus. Although *NahG* expressed the same level of ISR as Col-0 plants by *B. subtilis*, *sid2* mutant completely lost the resistance against *Pst* DC3000, suggesting that SA is also essential for *B. subtilis*-ISR against this pathogen (Figure 4C). However, *P. fluorescens* mounted equal level of ISR against both pathogens in *NahG* and *sid2* as Col-0, suggesting that *P. fluorescens*-ISR is independent on SA signaling pathway. In addition, both *P. fluorescens*- and *B. subtilis*-treated *npr1* mutants were more susceptible to *B. cinerea* and *Pst* DC3000 compared to bacteria-treated Col-0 plants (Figure 4A,C). However, bacteria-treated *npr1* plants, especially with *P. fluorescens*, appeared more resistant than non-treated mutant *npr1*. This suggests that *P. fluorescens*- and *B. subtilis*-triggered ISR is partially control by SA an NPR1, but other factors are also playing an important role in this immunity against *B. cinerea* and *Pst* DC3000.

The non-treated mutant *ein2.1* (Figure 4B) showed a high susceptibility to *B. cinerea* compared to non-treated Col-0. Furthermore, necrotic sizes were 1.5–2-times larger in *P. fluorescens-* and *B. subtilis*-treated *ein2.1*, respectively, compared to Col-0. This indicates that the ET signaling pathway is required for *P. fluorescens*- and *B. subtilis*-ISR against the necrotroph *B. cinerea*. Similarly, despite over 20% reduction of necrotic size of *B. cinerea* observed in bacteria-treated *jar1.2* plants, the ISR level triggered by *P. fluorescens* and *B. subtilis* against *B. cinerea* was not fully expressed in this mutant compared to Col-0, suggesting an important role of JA in regulating ISR by the two bacteria. Against *Pst* DC3000, the level of ISR induced by *P. fluorescens* and *B. subtilis* was completely discarded in *ein2.1* and *jar1.2* (Figure 4D). The percentage of diseased leaves of non-treated and bacteria-treated *ein2.1* and *jar1.2* was over 60% as observed in non-treated Col-0 plants. Data highlight a prominent role JA and ET signaling pathways in *P. fluorescens-* and *B. subtilis*-ISR against *Pst* DC3000.

#### 3.4.2. ABA Signaling Is Required for ISR against *Pst* DC3000, but not against *B. cinerea*

**Figure 5:**
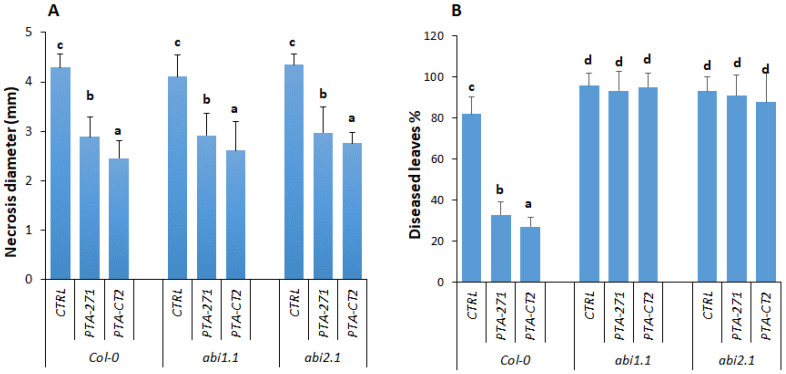
*B. subtilis* PTA-271 and *P. fluorescens* PTA-CT2-induced systemic resistance in Col-0 and its mutants *abi1.1* and *abi2.1* plants against *B. cinerea* and *Pst* DC3000. Three-week-old Col-0 and mutant plants *abi1.1* and *abi2.1* were pretreated with PTA-271 or PTA-CT2 suspensions for 2 weeks. Then the leaves were infected with *B. cinerea* and *Pst* DC3000. The ISR was determined at 4 dpi by measuring necrosis diameter caused by *B. cinerea* (**A**) and diseased leaves provoked by *Pst* DC3000 (**B**). Data are means ± SE from four independent experiments with a total of 16 plants/treatment (about 150 leaves). Different letters indicate statistically significant differences (Duncan test *p* < 0.05).

To understand whether ABA is involved in ISR against *Pst* DC3000 or *B. cinerea*, we compared the *P. fluorescens*- and *B. subtilis*-ISR effectiveness in Col-0 and its ABA-insensitive mutants *abi1.1* and *abi2.1*. Our data provided evidence that both *abi1.1* and *abi2.1* plants still expressed *P. fluorescens*- and *B. subtilis*-ISR against *B. cinerea* as in Col-0 plants (Figure 5A). The necrotic size provoked by *B. cinerea* in Col-0, *abi1.1*, and *abi2.1* remained comparable in both control and bacteria-treated plants. This indicates that ABA signaling was not required for ISR against *B. cinerea*, although ABA content was slightly increased by *B. subtilis* after *B. cinerea* infection. However, the loss of ABI1.1 and ABI2.1 function in the mutants strongly compromised ISR against *Pst* DC3000 in *P. fluorescens*- and *B. subtilis*-treated plants (Figure 5B). The mutant *abi1.1* and *abi2.1* showed around 90% of diseased leaves regardless bacterial treatment, while the percentage of diseased leaves in the bacteria-treated Col-0 was 3-fold lower than those in control plants, suggesting that both ABA concentration and signaling might be required for ISR against *Pst* DC3000.

#### 3.4.3. Loss of ABA Signaling in *abi1.1* and *abi2.1* Affects Defense Responses against Pathogens

**Figure 6:**
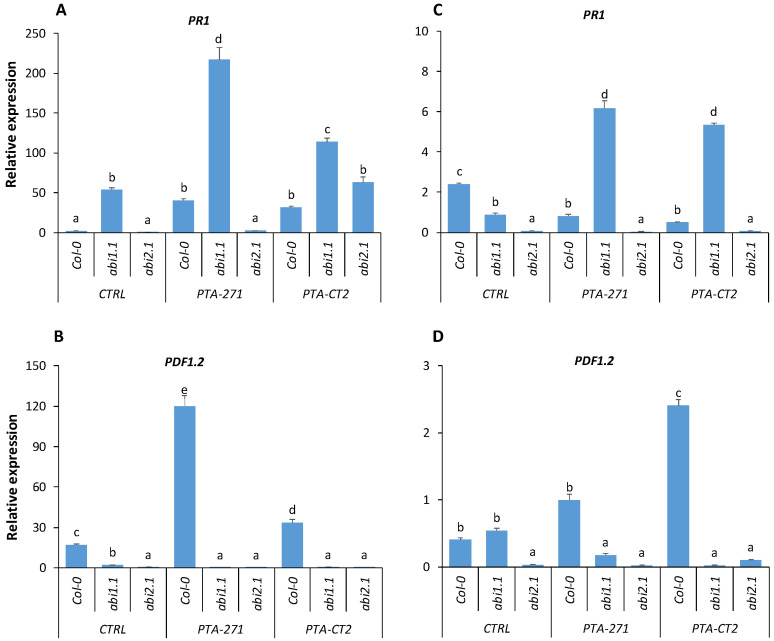
Expression of *PR1* and *PDF1.2* genes in leaves of control, *B. subtilis* PTA-271-, and *P. fluorescens* PTA-CT2-treated Col-0, *abi1.1*, and *abi2.1* after pathogen infection. The expression of *PR1* and *PDF1.2* genes was analyzed at 24 hpi with *B. cinerea* (**A**,**B**) and *Pst* DC3000 (**C**,**D**) in Col-0 and *abi1.1*, *abi2.1* plants. Data are mean ± SE from the pool of three independent experiments with 12 plants/treatment. Different letters indicate statistically significant differences (Duncan test, *p* < 0.05).

To investigate whether ABA signaling can affect immune response after pathogen infection, we compared the expression of *PR1* and *PDF1.2* genes in Col-0 and ABA insensitive mutants *abi1.1*, *abi2.1* after infection with *B. cinerea* and *Pst* DC3000. In *B. cinerea*-infected plants only *abi1.1* showed a high *PR1* expression compared to the control and *abi2.1* (Figure 6A). *B. subtilis* also failed to potentiate the expression of *PR1* in *abi2.1*, while in *abi1.1* mutant *PR1* was significantly upregulated after infection with *Pst* DC3000 (Figure 6B). Meanwhile, the enhanced expression of *PDF1.2* during *P. fluorescens*-ISR against *Pst* DC3000 was impaired in *abi1.1* and *abi2.1* plants (Figure 6D), highlighting a link between the strong reduction of *PDF1.2* expression in *P. fluorescens*-treated *abi1.1* and *abi2.1* and their susceptibility to *Pst* DC3000. Surprisingly, although the expression of *PR1* was strongly primed by *B. subtilis* in *abi1.1* and significantly reduced in *abi2.1* (Figure 6A), these mutants still expressed similar ISR level against *B. cinerea* as in Col-0 plants. Likewise, both *P. fluorescens* and *B. subtilis* were unable to increase expression of *PDF1.2* in *abi1.1* and *abi2.1* (Figure 6C), but no significant difference was observed in *P. fluorescens*- and *B. subtilis*-ISR against *B. cinerea* in Col-0, *abi1.1*, and *abi2.1* plants (Figure 2A).

#### 3.4.4. Root Treatment with Exogenous ABA Prior to Inoculation Has No Effect on ISR against *B. cinerea* and *Pst* DC3000

**Figure 7:**
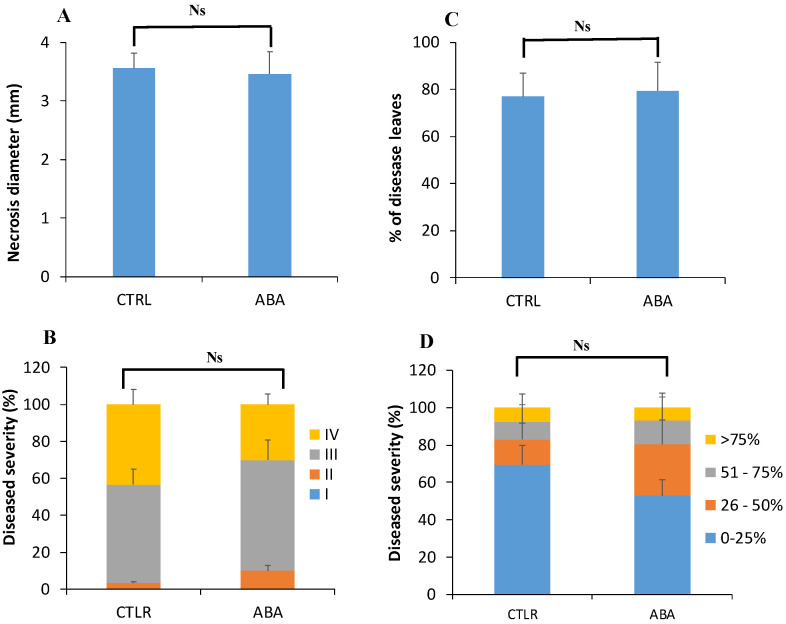
Effects of exogenous abscisic acid (ABA) on the induced resistance against *B. cinerea* and *Pst* DC3000 in Arabidopsis. Col-0 plants were treated with 100 µM ABA solution at the root level. After 48 h, the leaves were drop-inoculated with *B. cinerea* or sprayed with *Pst* DC3000. Disease incidence and severity caused by *B. cinerea* (**A**,**B**) and *Pst* DC3000 (**C**,**D**) were assessed at 4 days post infection. Data are mean ± SE from three repetitions with 12 plants/treatment. Ns indicates no significant difference (Duncan test, *p* < 0.05).

To test whether ABA effect is due to its concentration or not, exogenous ABA was applied at the root level of Col-0 plants and systemic resistance was assessed at 4 dpi with *B. cinerea* and *Pst* DC3000. Data showed that root treatment with ABA had no effect on systemic resistance against both pathogens. As for the control, ABA-treated plants displayed strong spread symptoms caused by *B. cinerea* (Figure 7A), and expressed approximately 80% of diseased leaves by *Pst* DC3000 (Figure 7B). No significant difference was observed in disease severity between control and ABA-treated plants upon *B. cinerea* and *Pst* DC3000 challenge (Figure 7C,D).

## 4. Discussion

In this study, we showed that *B. subtilis* PTA-271 and *P. fluorescens* PTA-CT2 are able to trigger an efficient protection against both the necrotrophic fungus *B. cinerea* and the hemibiotrophic bacterium *Pst* DC3000 in Arabidopsis. The effectiveness of *P. fluorescens*-ISR is mostly higher than *B. subtilis*-ISR against both pathogens. It is plausible to assume that different mechanisms could be involved in *P. fluorescens*- and *B. subtilis*-ISR against the pathogens. In both cases, ISR is based on priming immune response after perception of pathogen-derived signal, rather than a direct elicitation effect. This is in line with other reports indicating that ISR is linked to the priming plants for enhanced immune system only after pathogen challenge, saving the plant from a high energy consumption [6,7]. It is also suspected that *B. subtilis* and *P. fluorescens* prime at least partially distinct signaling defense pathways that result in different levels of ISR [5,8,13]. A clear difference between *B. subtilis*- and *P. fluorescens*-ISR is that they target distinct defense-related genes and signaling pathways depending on the pathogen. *P. fluorescens* significantly upregulated the expression of *PR1*, *PR4*, and *PDF1.2* at the early stage of *B. cinerea* infection, compared to *B. subtilis* that induced higher expression of *PDF1.2* at the later stage. This highlights the importance of earlier primed ET/JA-responsive defenses for enhanced efficiency of ISR against the necrotrophic fungus. The upregulation of *PR1* gene in bacteria-treated plants upon *B. cinerea* challenge suggests the dependency of ISR on the SA signaling pathway. This is in line with the findings of Nie et al. [13], showing that *B. cereus* AR156 primed *Arabidopsis* for enhanced expression of the *PR1* gene after *B. cinerea* infection. A substantially lower scale of *PR1* transcript was also observed compared to those of *PR4* and *PDF1.2* upon *B. cinerea* challenge, indicating the requirement of JA/ET-responsive defenses in ISR against this pathogen.

The phytohormone analysis suggests that beneficial bacteria differentially affect hormonal status before infection. *P. fluorescens* did not impact either ACC or ABA levels, but reduced SA and JA content in leaf tissues. *B. subtilis* also reduced JA level, while it induced a significant accumulation of ACC without impact on SA and ABA. It is therefore suggested that bacteria may confer a hormonal homeostasis, thus prime plants for enhanced resistance upon subsequent pathogen infection. It seems that the induced resistance by *P. fluorescens* was more dependent on JA/ET signaling rather than hormonal concentration, as reported earlier [53,54]. Although *P. fluorescens* did not cause any change in the amount of JA and ACC after *B. cinerea* infection, a significant increase of *PDF1.2* expression occurred. Meanwhile, *B. subtilis* induced an increase in JA content, and primed plants for the strongest expression of *PDF1.2* after challenge with *B. cinerea*. Interestingly, the *P. fluorescens*-ISR against *Pst* DC3000 is associated with enhanced expression of *PDF1.2*, but not of *PR1*, whereas *B. subtilis* primed plants for upregulation of *PR1*, but not *PDF1.2* after *Pst* DC3000 infection. In both cases, the expression of *PR4* was upregulated, indicating that ISR against the hemibiotrophic *Pst* DC3000 share similar ET-dependent signaling components [55]. It is noteworthy that *P. fluorescens* triggered a sooner and stronger expression of defense-related genes at early stage of *Pst* DC3000 infection, compared to *B. subtilis*. However, *B. subtilis* activated later a high expression of *PR1* and *PR4* genes, indicating the prominent role of SA-dependent immune response in ISR against *Pst* DC3000, as also observed with *B. cereus* and *P. fluorescens* SS101 [14,16,25]; *B. subtilis* PTA-271 also increased the amounts of SA and JA after *Pst* DC3000 infection, suggesting that this beneficial bacterium can also modulate SA-JA crosstalk by prioritizing SA-dependent immune response after *Pst* DC3000 infection, as observed with *P. fluorescens* SS101 (van de Mortel et al., 2012). It has also been reported that *Pst* DC3000 can produce the phytotoxin coronatine, which mimics a bioactive JA conjugate and targets JA-receptor COI1, resulting in an activation of JA-dependent response and suppression of SA-inducible defense response [56,57,58].

Functional analyses also point to the involvement of SA signaling in *B. subtilis*-ISR, but not in *P. fluorescens*-ISR against *B. cinerea*, since mutant *sid2* and transgenic *NahG* plants (both SA deficient) failed to express the *B. subtilis*-ISR, but not *P. fluorescens*-ISR against *B. cinerea*, compared to Col-0. Similarly, *B. subtilis*-mediated ISR against *Pst* DC3000 was abolished in *sid2* mutant but not in *NahG* plants, suggesting that SA is involved in *B. subtilis*-ISR against this bacterium. The phenotypic difference between *NahG* and *sid2* after *Pst* DC3000 infection could be related to different basal level of SA in these mutants [59], since *NahG* plants express a SA hydrolase to degrade SA to catechol, while *sid2* is unable to synthesize SA due to lack of isochorismate synthase 1. Our data are also consistent with the finding that ISR triggered by *Paenbacillus alvei K165* was impaired in SA-signaling *eds5* and *sid2* mutants, but still fully expressed in *NahG* transgenic plants [60]. In contrast, *P. fluorescens* triggered SA-independent ISR against *Pst* DC3000. This highlights a distinct dependency of SA signaling by *P. fluorescens* and *B. subtilis* to mediated ISR against both necrotroph and hemibiotroph pathogens. It has been reported that in Arabidopsis, SA can serve as a precursor for synthesis of SA-containing siderophores by rhizospheric bacteria in iron-limiting conditions [61,62]. ISR induced by several Bacillus strains also required the SA signaling pathway [63].

We also showed that both *P. fluorescens* and *B. subtilis* failed to mediate ISR against *B. cinerea* and *Pst* DC3000 in *npr1*, *jar1.2*, and *ein2.1* mutants. This result is consistent with most studies, highlighting the functional role of JA/ET and NPR1 in ISR triggered by both beneficial bacteria against the pathogens. This also suggests a prominent function of NPR1 in SA-dependent or independent ISR [5]. *NPR1* functions downstream with SA- and JA/ET-signaling pathways, leading to activation of transcription factors and defense-related genes, such as *PR1* and *PDF1.2* [20]. SA- and JA/ET-dependent ISR induced by *B. cereus* AR156 required NPR1 after *Pst* DC3000 infection [16]. In *B. subtilis* PTA-271-treated plants, NPR1 might play a role as a mediator of SA-JA crosstalk, resulting in priming both SA- and ET/JA-responsive genes following infection with *B. cinerea*. Our results showed that *ein2.1* and *jar1.2* mutants were unable to express a full ISR mediated by *P. fluorescens* PTA-CT2 and *B. subtilis* PTA-271 against *B. cinerea* and *Pst* DC3000. However, roles of JA and ET signaling seem to be more prioritized in the ISR against *Pst* DC3000.

The role of ABA in priming plant immune response by beneficial microbes is so far obscure and even unknown. Here, we showed that the amount of ABA was significantly increased in *B. subtilis* PTA-271-treated plants after infection with both *B. cinerea* and *Pst* DC3000, while it was accumulated only in *P. fluorescens* PTA-CT2-ISR against *Pst* DC3000, but not *B. cinerea*. This indicates that ABA can play a positive role in ISR depending on beneficial strain and pathogen lifestyle. ABA can act downstream SA signaling to suppress stomatal reopening induced by pathogens, inhibiting pathogenic penetration [28,30,64,65]. *Pst* DC3000-induced stomatal reopening was strongly suppressed by *B. subtilis* FB17, thus minimizing the bacteria entry [28]. The increase of ABA level during *B. subtilis*-ISR could be related to its interaction with the SA signaling pathway upon *Pst* DC3000 infection [14,15,16]. Interestingly, a significant difference was observed between *P. fluorescens* PTA-CT2 and *B. subtilis* PTA-271 in inducing ABA accumulation after *B. cinerea* infection, suggesting that ABA concentration can contribute to ISR against the pathogens. Moreover, before pathogen infection the higher level of ABA in *P. fluorescens*- and *B. subtilis*-treated plants did not seem to play such an important role for the subsequent ISR against pathogens. This suggests that *P. fluorescens* and *B. subtilis* are likely to modulate interaction between ABA and SA or ET/JA signaling pathways, thus abolishing the negative effect of ABA in ISR against pathogens.

Using ABA-insensitive mutants, we showed that ABA plays predominant role in regulating ISR against *Pst* DC3000, but not *B. cinerea*. Loss of ABA function in *abi1.1* and *abi2.1* plants did not cause any consistent change in ISR level against *B. cinerea*. This suggests that both *P. fluorescens*- and *B. subtilis*-ISR share the ABA-independent pathway, albeit these bacteria induced different levels of ABA accumulation upon *B. cinerea* challenge. However, ABA was found to be determinant for both *P. fluorescens*- and *B. subtilis*-mediated ISR against *Pst* DC3000. Mutation of ABA signaling in *abi1.1* and *abi2.1* results in impaired ISR by both bacteria toward *Pst* DC3000. This is in line with the demonstration that inhibition of ABA signaling can cause stomatal reopening induced by *Pst* DC3000 [30,34], which is essential in early stages of pathogenic penetration [34,36]. Since the *abi* mutants actually do contain normal amounts of ABA as the wild type, it is conceivable that other ABA signaling components are involved in ISR including independent modulation of the guard cell outward-rectifying potassium channel (GORK) [66]. Such a mechanism may implicate ABA in the direct and rapid stomatal responses to the onset of pathogenic infection. Further investigations are needed to decipher the ABA signaling components in the regulation of priming immune response. It is suggested that both *P. fluorescens* and *B. subtilis* require ABA signaling at least via the ABI receptor for ISR against the hemibiotroph *Pst* DC3000 rather than the necrotroph *B. cinerea*. Experiments showed that the loss BI2.1 function, but not of ABI1.1, compromised the expression of *PR1*, resulting in a reduction of *B. subtilis*-ISR against *Pst* DC3000. This effect may be linked to a significant downregulation of JA/ET-responsive *PDF1.2* gene, supporting the role of JA/ET in ISR against pathogens [5,13,14]. However, exogenous application of ABA at the root level did not show any significant change in ISR induced by both bacteria, suggesting that ISR is more sensitive to ABA signaling rather than ABA concentration.

Overall, this study clearly reports that *B. subtilis* PTA-271 and *P. fluorescens* PTA-CT2 prime common and distinct immune responses resulting in differential effectiveness of ISR against pathogens. *P. fluorescens* seems to potentiate rapid and strong defense responses at the early stage of the pathogen infection, triggering an efficient resistance compared to *B. subtilis*. Both *B. subtilis*- and *P. fluorescens*-ISR share JA/ET and NPR1-dependent defenses as prioritized immune pathways against *B. cinerea*. However, *B. subtilis*-ISR against *Pst* DC3000 is dependent on SA, JA/ET, and NPR1 pathways, while *P. fluorescens*-ISR is independent on SA pathway. ABA signaling, but not ABA concentration, also plays an important role along with JA/ET signaling in primed systemic immunity by beneficial bacteria against *Pst* DC3000, but not against *B. cinerea*.

## Supplementary Materials

The following are available online at https://www.mdpi.com/2076-393X/8/3/503/s1, Table S1: Primer sequences of the different genes used in this study.
