# Supplementary material for: Bacillus subtilis and Pseudomonas fluorescens Trigger Common and Distinct Systemic Immune Responses in Arabidopsis thaliana Depending on the Pathogen Lifestyle
Source: Vaccines (Basel). 2020 Sep 4;8(3):503. doi: 10.3390/vaccines8030503 (PMC7563191; doi:10.3390/vaccines8030503)
Supplement: Supplementary file 1 [file vaccines-08-00503-s001.pdf]

Supplementary material

Table S1: Primer sequences of the different genes used in this study

**Genes**	**Locus**	**Forward Primer (5′ – 3′)**	**Reverse Primer (3′ – 5′)**
PR1	AT2G14610	TCTTCCCTCGAAAGCTCAAG	AAGGCCCACCAGAGTGTATG
PR4	AT3G04720	AACAATGCGGTCGTCAAGGC	AAGCACTCACGGCTCTCAAATCCC
PDF1.2	AT5G44420	TCACCCTTATCTTCGCTGCT	GTTGCATGATCCATGTTTGG
UBQ5	AT3G62250	GGAAGAAGAAGACTTACACC	AGTCCACACTTACCACAGTA
